# Low-frequency maternal novel MYH7 mosaicism mutation in recurrent fetal-onset severe left ventricular noncompaction: a case report

**DOI:** 10.3389/fped.2023.1195222

**Published:** 2023-06-08

**Authors:** Hiroshi Kawamura, Masamichi Ikawa, Keiichi Hirono, Junya Kimura, Takashi Okuno, Masao Kawatani, Kunihiro Inai, Yukiko Hata, Naoki Nishida, Yoshio Yoshida

**Affiliations:** ^1^ Department of Obstetrics and Gynecology, University of Fukui, Fukui, Japan; ^2^ Department of Medical Genetics, University of Fukui Hospital, Fukui, Japan; ^3^ Department of Pediatrics, University of Toyama, Toyama, Japan; ^4^ Division of Diagnostic Pathology, Surgical Pathology, University of Fukui Hospital, Fukui, Japan; ^5^ Department of Pediatrics, University of Fukui, Fukui, Japan; ^6^ Department of Molecular Pathology, University of Fukui, Fukui, Japan; ^7^ Department of Legal Medicine, Faculty of Medicine, University of Toyama, Toyama, Japan

**Keywords:** left ventricular noncompaction, myosin heavy chain 7, next-generation sequencing, mosaicism mutation, case report

## Abstract

**Background:**

Left ventricular noncompaction (LVNC) is a rare inherited cardiomyopathy with a broad phenotypic spectrum. The genotype-phenotype correlations in fetal-onset LVNC have not yet been fully elucidated. In this report, we present the first case of severe fetal-onset LVNC caused by maternal low-frequency somatic mosaicism of the novel myosin heavy chain 7 (MYH7) mutation.

**Case presentation:**

A 35-year-old pregnant Japanese woman, gravida 4, para 2, with no significant medical or family history of genetic disorders, presented to our hospital. In her previous pregnancy at 33 years of age, she delivered a male neonate at 30 weeks of gestation with cardiogenic hydrops fetalis. Fetal echocardiography confirmed LVNC prenatally. The neonate died shortly after birth. In the current pregnancy, she again delivered a male neonate with cardiogenic hydrops fetalis caused by LVNC at 32 weeks of gestation. The neonate died shortly after birth. Genetic screening of cardiac disorder-related genes by next-generation sequencing (NGS) was performed which revealed a novel heterozygous missense MYH7 variant, NM_000257.3: c.2729A > T, p.Lys910Ile. After targeted and deep sequencing by NGS, the same MYH7 variant (NM_000257.3: c.2729A > T, p.Lys910Ile) was detected in 6% of the variant allele fraction in the maternal sequence but not in the paternal sequence. The MYH7 variant was not detected by conventional direct sequencing (Sanger sequencing) in either parent.

**Conclusions:**

This case demonstrates that maternal low-frequency somatic mosaicism of an MYH7 mutation can cause fetal-onset severe LVNC in the offspring. To differentiate hereditary MYH7 mutations from *de novo* MYH7 mutations, parental targeted and deep sequencing by NGS should be considered in addition to Sanger sequencing.

## 1. Introduction

Left ventricular noncompaction (LVNC) is a rare inherited cardiomyopathy characterized morphologically by a severely thickened two-layered myocardium, excessive trabeculation of the left ventricle, and deep intertrabecular recesses leading to the left ventricular cavity (1). LVNC has a wide range of phenotypic expressions, ranging from severe prenatal manifestations to asymptomatic presentation in adulthood (2). The condition has been shown to have either a sporadic or familial genetic background, with hereditary causes accounting for approximately 30% of all LVNC cases (3). Recent studies using large cardiac disease gene panels have identified several genetic variants responsible for LVNC (4, 5). Of the genetic mutations causing LVNC, more than 50% are associated with sarcomeres, which are the smallest functional unit of the striated muscle tissue. The most common sarcomere-related gene associated with LVNC is that encoding for myosin heavy chain 7 (MYH7) (5).

With recent advances in genetic analysis technology, some genetic variants originally thought to be *de novo* mutations have been shown to be caused by parental somatic or gonadal mosaic mutations (6–8). Recent studies revealing parental mosaicism emphasize the importance of using more sensitive analysis techniques, such as digital polymerase chain reaction (dPCR), pyrosequencing, high-resolution melting analysis, and next-generation sequencing (NGS), rather than conventional direct sequencing (i.e., Sanger sequencing). The detection of parental mosaic MYH7 mutations associated with familial LVNC using sensitive methods has not yet been reported.

Herein, we report an extremely rare case of recurrent non-immune hydrops fetalis caused by fetal-onset severe LVNC associated with a mosaic mutation in the MYH7 gene.

## 2. Case presentation

**Figure 1:**
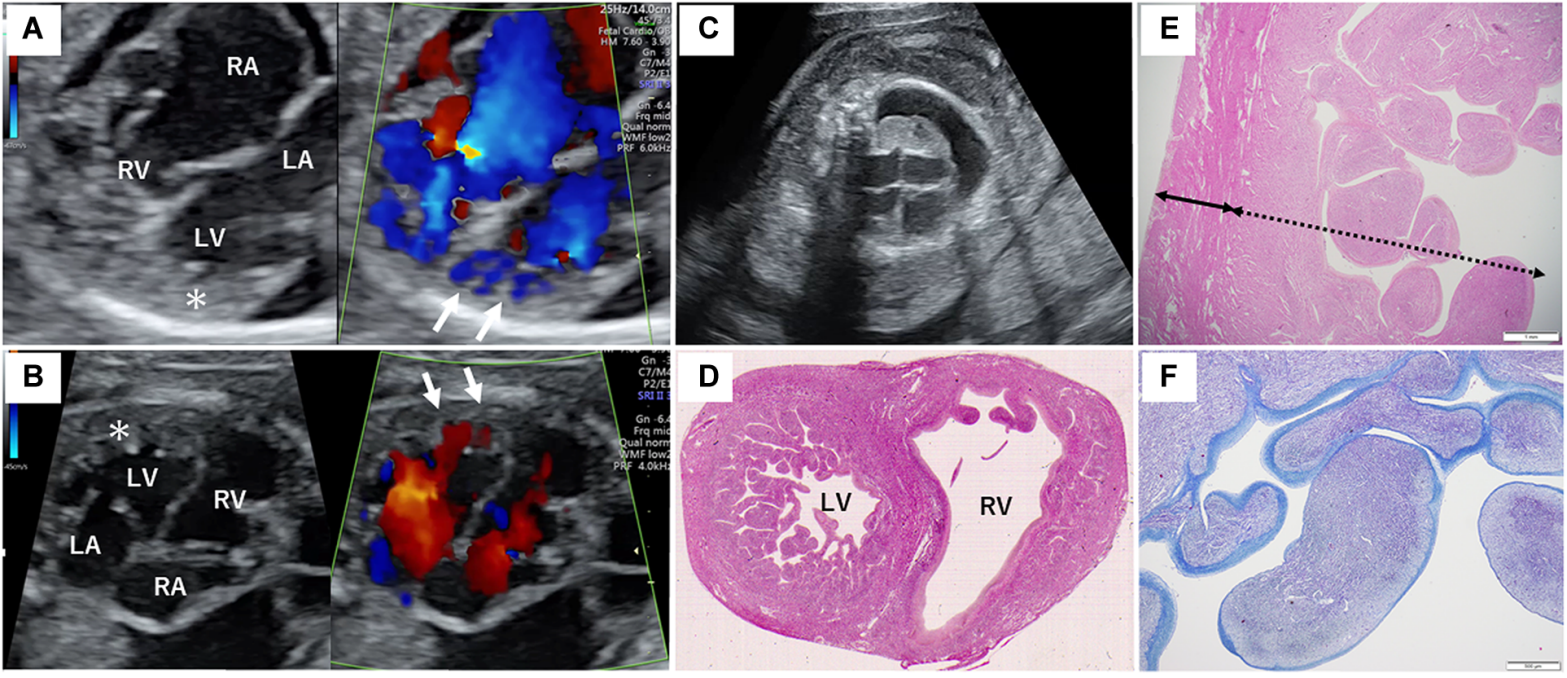
Fetal sonography and pathological findings of the proband's heart. (**A**) Fetal echocardiography at 26 + 0 weeks of gestation during the second pregnancy. (**B**) Fetal echocardiography at 25 + 4 weeks of gestation during the third pregnancy. The asterisk indicates the severely thickened myocardium, and the white arrows show the blood flow into the deep intertrabecular recesses of the left ventricle. (**C**) Fetal sonography showing marked subcutaneous edema and right pleural effusion in the proband. (**D**) Hematoxylin and eosin staining of the horizontal section of the middle level of the ventricular long axis of the autopsy. Remarkable trabeculation of the left ventricle is detected. (**E**) The solid bidirectional arrow shows the compacted layer, and the dotted bidirectional arrow shows the thickened non-compacted layer of the left ventricle. (**F**) Histological appearance with Azan staining reveals fibrosis of the endocardium of the left ventricle. The endocardium is stained blue more intensely. RA, right atrium; RV, right ventricle; LA, left atrium; LV, left ventricle.

A 35-year-old pregnant Japanese woman (spontaneously conceived), gravida 4, para 2, with no significant medical or family history, presented to our hospital. She had a spontaneous abortion during the first trimester of her first pregnancy. In the second pregnancy at 28 years of age, she delivered a healthy term female newborn with a birthweight of 2,908 grams. In the third pregnancy at 33 years of age, she was referred to our hospital at 23 + 5 weeks of gestation for perinatal management of hydrops fetalis. The fetus presented with remarkable systemic edema, ascites, and bilateral pleural effusion on transabdominal ultrasonography. Detailed fetal echocardiography revealed significant cardiomegaly (cardiothoracic area ratio, 56.7%) with severe tricuspid valve regurgitation (TR) and moderate mitral valve regurgitation (MR). Both ventricles were hypokinetic; the left and right myocardial performance indices were 0.92 and 0.85, respectively. Non-compacted layers in both ventricles and an extensive trabeculated layer with multiple deep intertrabecular recesses filled with blood directly from the left ventricular cavity were identified. Fetal echocardiography at 26 + 0 weeks of gestation is shown in Figure 1A. The fetus was diagnosed with cardiogenic hydrops fetalis caused by LVNC. At 30 + 6 weeks of gestation, regular uterine contractions with cervical dilation occurred spontaneously, indicating the onset of labor. We discussed the mode of delivery with the parents and decided on vaginal delivery because of the fatal prognosis of the fetus. At 31 + 0 weeks of gestation, the mother delivered a male neonate with a birth weight of 2,440 grams; Apgar scores of 1 and 1 at 1 and 5 min, respectively; and umbilical arterial pH of 7.310. The newborn died soon after birth, despite neonatal resuscitation. We offered the parents a pathological autopsy and genetic testing of the newborn to obtain important information for future pregnancies; however, they declined these examinations.

**Figure 2:**
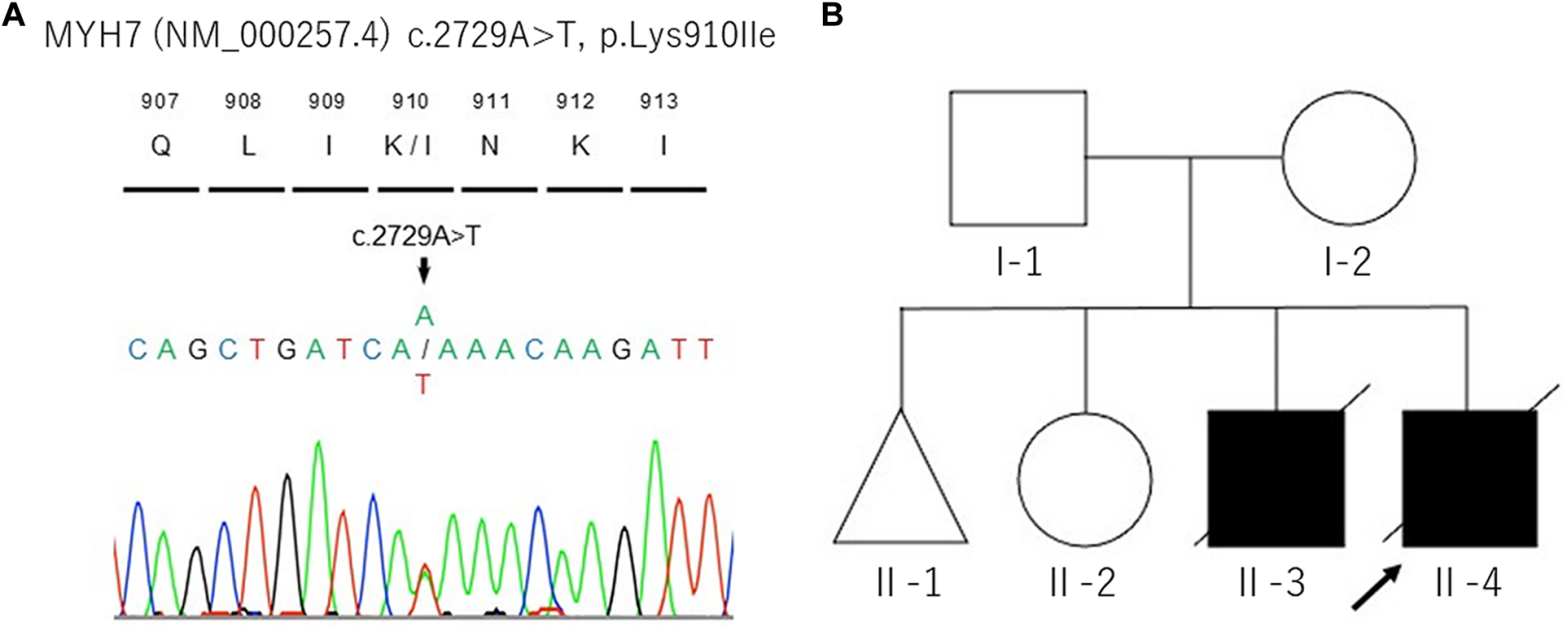
DNA sequencing analysis of proband's whole blood (**A**) and the pedigree of this family (**B**). The black arrow indicates the proband (II-4).

**Figure 3:**
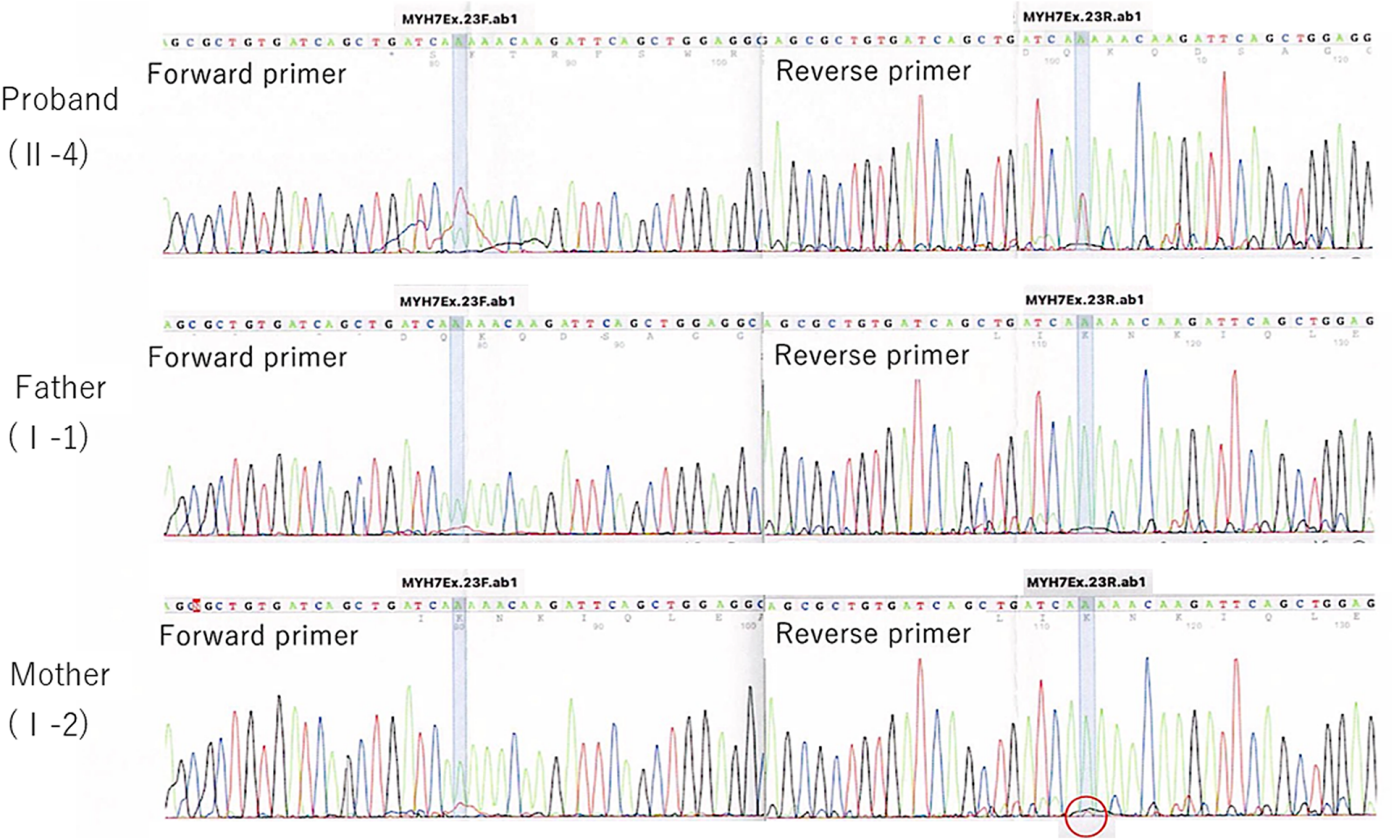
Direct sequencing trio analysis chromatograms. A red circle indicates a slight change (c.2729A > T) of unclear significance.

**Figure 4:**
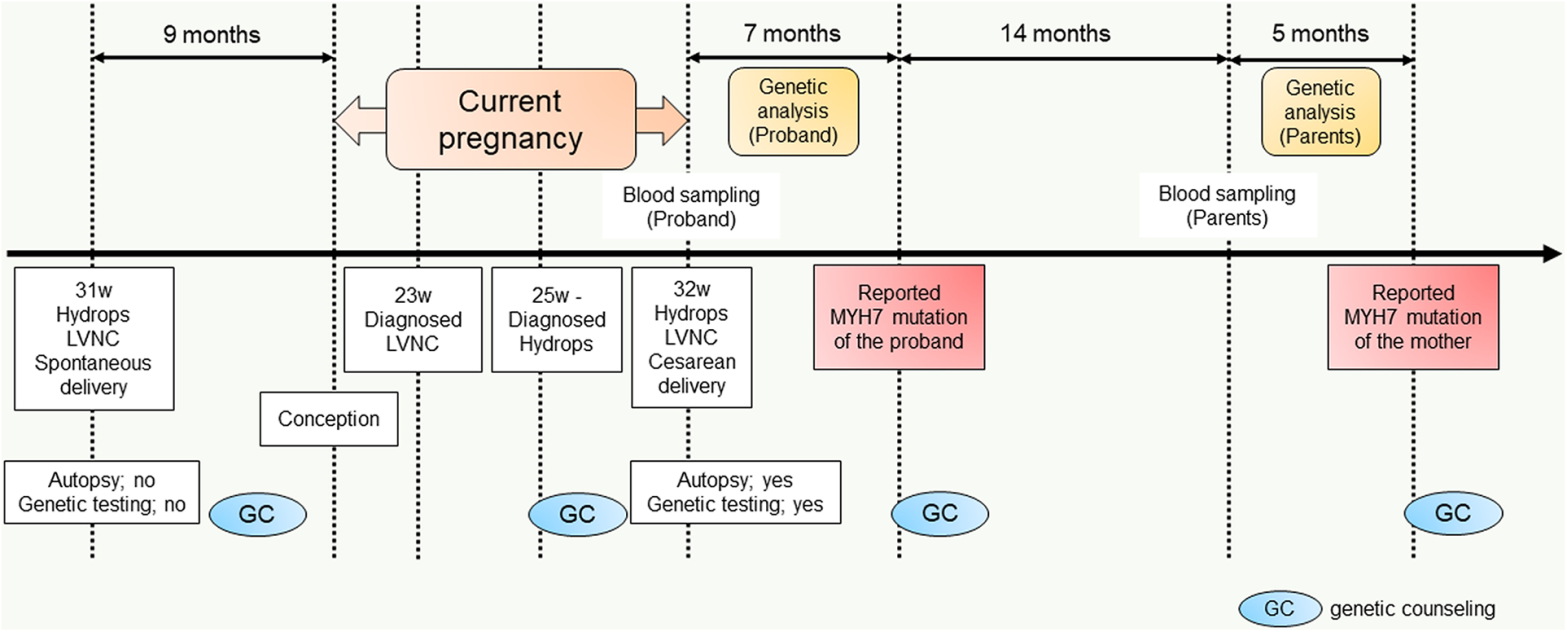
Diagram of the workflow from the delivery of the previous fetus (II-3) to parental sequencing.

In the current pregnancy, we monitored the fetus every 2–3 weeks after 13 weeks of gestation, especially for the fetal hydropic sign and contractile dysfunction of the fetal heart on ultrasound. The mother did not take any precautions or interventions because there was no effective prevention of recurrence of hydrops fetalis. At 21 + 1 weeks of gestation, abnormal findings were not detected. However, at 23 + 1 weeks of gestation, we detected noticeably decreased ventricular contraction with moderate TR and MR. In addition, echocardiographic findings observed in the fetal myocardium were consistent with LVNC and were almost similar to those in the previous fetus. At 25 + 4 weeks of gestation, hydropic signs including systemic skin edema, pleural effusion, and ascites were noted. Fetal echocardiography is shown in Figure 1B. The fetal course was very similar to that of the previous fetus; therefore, we strongly suspected familial LVNC. The hydropic signs worsened during the course of the pregnancy (Figure 1C). Based on the previous pregnancy, we discussed perinatal management (especially the timing and mode of delivery) with the parents and respecting the parents’ wishes, decided to perform cesarean section to prevent fetal death. There were no maternal cardiac signs suggestive of Mirror syndrome. At 31 weeks of gestation, transabdominal sonography revealed an edematous and thickened placenta, and pulse-wave Doppler revealed continuous absent or reversed blood flow in the umbilical artery. To avoid fetal death, a cesarean section was performed at 32 + 0 weeks of gestation. A male newborn with breech presentation was delivered. His birth weight was 2,680 grams; Apgar scores at 1 and 5 min were 1 and 1, respectively; and umbilical arterial pH was 7.194. The newborn died shortly after birth, despite neonatal resuscitation. The placenta was grossly edematous, weighing 760 grams. The mother's postoperative course was favorable, and she was discharged without complications. In contrast to the previous pregnancy, the parents requested a pathological autopsy and genetic testing of the neonate. Gross anatomical findings at autopsy included systemic skin edema, bilateral pleural effusion, ascites, markedly dilated right ventricle, and hypertrophic left ventricle. Histological examination of the left ventricle revealed a two-layered structure composed of a prominent trabeculated and compacted layer (Figures 1D, E), and endocardial elastic fibrous proliferation throughout the left ventricle (Figure 1F). After obtaining informed consent from the parents, DNA was isolated from the neonatal whole blood. Genetic screening of 182 cardiac disorder-related genes associated with cardiomyopathies and channelopathies by NGS revealed a novel heterozygous missense MYH7 variant, NM_000257.3: c.2729A > T, p.Lys910Ile, which had not been reported in several genetic databases, including ClinVar, HGMD®, dbSNP, gnomAD, and Jmorp (Japanese Multi Omics Reference Panel) (Figure 2A). The GADD score and PolyPhen-2, which predicted the effect of a variant on protein function, showed that this novel MYH7 variant was pathogenic. The pedigree is shown in Figure 2B. After genetic counseling, parental genetic analyses were performed. By Sanger sequencing, the MYH7 variant was not detected in the father (Ⅰ-1), while in the mother (Ⅰ-2) a slight change (c.2729A > T) of unclear significance was detected in the chromatogram (Figure 3). Therefore, targeted and deep sequencing was performed on the whole blood of both parents by NGS. The same MYH7 variant (NM_000257.3: c.2729A > T, p.Lys910Ile) was detected in 6% (807/14,266 coverages) of the variant allele fraction (VAF) in the maternal sequence but not in the paternal sequence [0% (0/1,658 coverages) of VAF]. Echocardiography revealed no abnormal findings in the myocardia of the parents. The parents did not present with generalized muscle weakness. Furthermore, there was no increase in the maternal serum creatine kinase. Figure 4 illustrates the workflow from the delivery of the previous fetus (II-3) to parental sequencing.

## 3. Discussion and conclusions

To the best of our knowledge, this is the first report of fetal-onset severe LVNC caused by maternal low-frequency somatic mosaicism of the novel MYH7 mutation.

The MYH7 gene is composed of 40 exons of approximately 23 kilobases and is located on the long arm of chromosome 14 (14q11.2-q13). It encodes myosin heavy chain (MHC)-β and slow MHC, which are expressed in the myocardium and type 1 skeletal muscle fibers, respectively (9). A pathogenic MYH7 mutation was first reported by Lowrance et al. in patients with familial hypertrophic cardiomyopathy (10). Currently [HGMD®, available at http://www.hgmd.org (accessed February 11, 2023)], more than 1,200 MYH7 variants have been classified as pathogenic gene mutations.

To date, there have been few reports of mosaic mutations in MYH7. Forissier et al. reported two siblings with familial hypertrophic cardiomyopathy that was probably caused by maternal germline MYH7 mosaicism (11). In another study, somatic MYH7 mosaicism was identified in the father of the proband who had distal myopathy. In that report, the mosaicism was evaluated in the paternal peripheral blood cells (12). However, these two reports did not describe the VAF of MYH7 variants. In the present case, the targeted and deep sequencing by NGS was effective in detecting the rather low frequency (6.0%) mosaicism of the maternal MYH7 mutation, which was unclear by Sanger sequencing. In a previous report, the detection limit of Sanger sequencing was reported to be 15%–20% of the VAF (13). Our case report shows that the detection of the rather low-frequency mosaicism, which was undetectable by the conventional analysis method, was attributed to obtaining more accurate information about parental mosaicism.

The heterozygous missense MYH7 variant detected in both the proband (II-4) and his mother (I-2) is a novel variant that has not been previously reported in the available genetic databases. We judged this variant to be the pathogenic mutation causing LVNC with hydrops fetalis in the proband (II-4), based on the GADD score and PolyPhen-2 results. The similarity in the phenotypes of the proband and those of the previous fetus (II-3), including fetal-onset of heart failure and echocardiographic findings characteristic of LVNC, suggested that the same variant was probably present in the two brothers.

A recent systematic review identified 66 genes responsible for LVNC, and found that sarcomere-related genes, including MYH7, accounted for 52% of LVNC causal genes (5). Interestingly, this review showed that the risk of major adverse cardiac events was relatively low in patients, especially adults, with sarcomere-related gene mutations. Furthermore, among the sarcomere-related genes, MYH7 was associated with the lowest risk of major adverse cardiac events. However, in the present report, the MYH7 mutation caused progressive heart failure in the neonatal proband.

MYH7 mutations play an important role in the development of severe heart failure in fetal-onset LVNC. Previous reports of MYH7-related fetal LVNC found heart failure at mid to late of 2nd trimester of pregnancy (14, 15). In the proband (II-4) of our case, a fetal echocardiography showed signs of LVNC at 23 weeks of gestation and hydropic sign at 25 weeks of gestation, which is consistent with the clinical characteristics of previous reports. A study of the genetic background of 33 cases of fetal-onset LVNC showed that the most frequent causative variants were detected in MYH7 (7 out of 15 variants) (16). The reported variants were widely distributed between exon 5 and exon 37 of MYH7, and the present case harbored a variant in exon 23 of MYH7. Moreover, MYH7 variants cause hypertrophic and dilated cardiomyopathy in addition to LVNC, suggesting that the location of the variants may not be associated with the LVNC phenotype. Few studies have demonstrated MYH7 variants in fetal-onset LVNC (16, 17); therefore, further investigations are needed to validate the genotype and phenotype correlations in fetal-onset LVNC in more detail.

A strength of this report is that it highlights the importance of distinguishing true *de novo* variants from inherited, low-mosaic mutations in the diagnosis of genetic diseases, which probably contributes to assessment of the recurrence risk. One of the limitations of this report is that in the present case, MYH7 mosaicism was evaluated only in the peripheral blood cells and not in the germline cells because it was impossible to obtain germline cells from the mother without an invasive procedure. Another limitation of this report is that the possibility that unknown genetic mutations which not included in the screening of 182 cardiac-disorder related genes in this study may be involved.

In conclusion, this case demonstrates that maternal low-frequency somatic mosaicism of an MYH7 mutation can cause fetal-onset severe LVNC in the offspring and that parental targeted and deep sequencing by NGS should be considered to differentiate hereditary mutations from *de novo* mutations. This important finding should contribute to the genetic counseling of parents who wish to elucidate the genetic causes of recurrent severe perinatal adverse outcomes in their offspring.

## Data Availability

The datasets presented in this study can be found in online repositories. https://www.ncbi.nlm.nih.gov/nuccore/LC765465. Further inquiries can be directed to the corresponding author.
